# Comparison of SHAPE reagents for mapping RNA structures inside living cells

**DOI:** 10.1261/rna.058784.116

**Published:** 2017-02

**Authors:** Byron Lee, Ryan A. Flynn, Anastasia Kadina, Jimmy K. Guo, Eric T. Kool, Howard Y. Chang

**Affiliations:** 1 Center for Personal Dynamic Regulomes, Stanford University, Stanford, California 94305, USA; 2 Department of Chemistry, Stanford University, Stanford, California 94305, USA

**Keywords:** SHAPE, RNA structure

## Abstract

Recent advances in SHAPE technology have converted the classic primer extension method to next-generation sequencing platforms, allowing transcriptome-level analysis of RNA secondary structure. In particular, icSHAPE and SHAPE-MaP, using NAI-N_3_ and 1M7 reagents, respectively, are methods that claim to measure in vivo structure with high-throughput sequencing. However, these compounds have not been compared on an unbiased, raw-signal level. Here, we directly compare several in vivo SHAPE acylation reagents using the simple primer extension assay. We conclude that while multiple SHAPE technologies are effective at measuring purified RNAs in vitro, acylimidazole reagents NAI and NAI-N_3_ give markedly greater signals with lower background than 1M7 for in vivo measurement of the RNA structurome.

## INTRODUCTION

RNA molecules have the inherent ability to fold into complex structures through base-pairing and three-dimensional interactions, allowing them to interact with all types of molecules, while still containing genetic information (Sharp 2009). For decades biochemists have used chemical probes as reagents to study the structure of RNA molecules to infer how structure contributes to function. Chemical probing of RNA structure has emerged as one of the most widely used methods in molecular biology, with many laboratories around the world using it to study RNA (Weeks 2010; Kubota et al. 2015).

Dimethylsulfate (DMS) RNA methylation, for example, is used to identify base-paired adenosine and cytosine nucleotides (Peattie and Gilbert 1980). DMS has been used both inside and outside the cell to study RNA structure, RNA–protein interactions, and even RNA structure changes that result in ligand binding (Kwok et al. 2013; Rouskin et al. 2014; Fang et al. 2015). Although very useful, DMS suffers from the drawback of having nucleotide specificities, limiting its resolution as a general RNA structure probe.

In contrast, SHAPE (selective 2′-hydroxyl acylation and primer extension) is unique in that it interrogates all four nucleobases, enabling structure measurements at single-nucleotide resolution. Furthermore, there has been a recent expansion of SHAPE to the intracellular context. For the most part, this has been the result of novel chemical and biochemical designs that enable robust RNA modification inside cells. For these reasons, SHAPE is now regarded as a gold standard chemical strategy to measure RNA secondary structure (Weeks and Mauger 2011). Chemically, SHAPE reagents measure RNA structure by selective acylation of the 2′ hydroxyl of accessible or flexible ribonucleotides, which predominantly occur in single-stranded regions in RNAs. As with most chemical methods to probe RNA structure, the SHAPE reagent adduct blocks reverse transcriptase (RT) elongation at the modified base. As such, the ability to identify the sites of RT-stops in the cDNA is critical for the accuracy of all structure measurements made by SHAPE. Traditionally, these truncated cDNA fragments are read out directly by denaturing gel electrophoresis.

**FIGURE 1.**
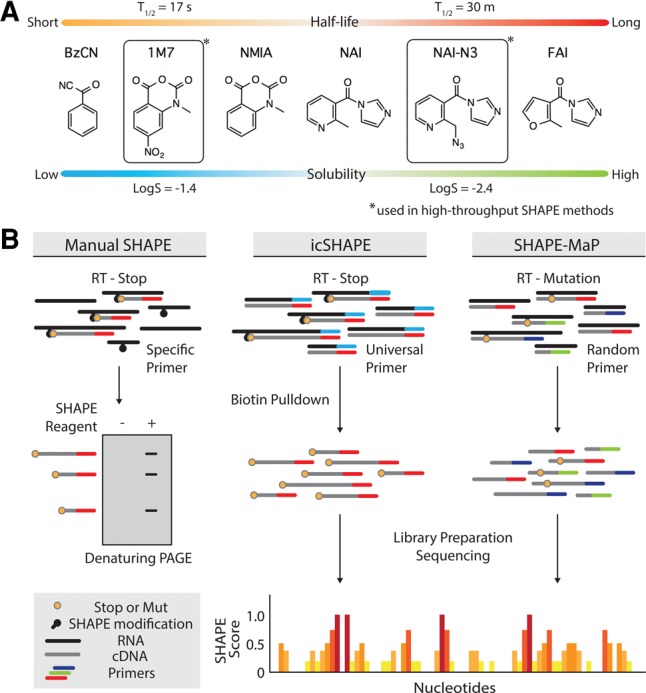
Overview of SHAPE reagents and experimental strategies. (*A*) Graphic representing the relative half-life and solubilities of SHAPE reagents, which are used for a variety of RNA structure probing methods, including icSHAPE and SHAPE-MaP. Actual structures are shown. (*B*) Schematic of various methods that take advantage of SHAPE electrophile chemistry. Key steps and differences between each method are shown in the figure.

More recently, conversion of cDNA fragments into sequencing libraries has enabled high-throughput sequencing methods for studying RNA secondary structure. In vivo CLICK SHAPE (Spitale et al. 2015) and SHAPE and mutational profiling (Siegfried et al. 2014) are two recently developed methods that use SHAPE chemistry to read out RNA structure information. Collectively, these new sequencing-based SHAPE technologies have opened the door for scientists to make single-nucleotide resolution measurements of RNA structure across entire transcriptomes and full-length RNA molecules (Siegfried et al. 2014; Spitale et al. 2015; Watters et al. 2016). However, they differ at many key levels. First, icSHAPE leverages a newer SHAPE electrophile NAI-N_3_, while SHAPE-MaP uses 1M7 or NMIA (Fig. 1A). These reagents differ markedly in solubility and reactivity. Second, icSHAPE relies on cDNA truncations while SHAPE-MaP relies on cDNA mutation (Fig. 1B). Third, icSHAPE sequencing libraries are enriched for structure informative (i.e., acylated) molecules, while SHAPE-MaP sequences all generated cDNA fragments regardless of whether they have been acylated (Fig. 1B). Fourth, published data for each experimental type were produced in different cell types from differentially purified RNA inputs. Despite these substantial differences, a recent report compared these two methods (Smola et al. 2015) without attempting to control for the aforementioned differences in SHAPE reagents and method, and without direct experimental testing of the two methods.

Differences in RNA structure data between traditional denaturing gel electrophoresis and sequencing may arise at any point from library construction to data analysis. Perhaps most importantly, the signal-to-noise ratio of RNA modification is likely to make a large difference, because it is the raw measurement of RNA structure. In an attempt to fill the lack of direct comparisons between current SHAPE methods with primary data, we report here a simple, but crucial comparison between SHAPE electrophiles. We generated primary data consisting of denaturing gel electrophoresis to demonstrate that the newly designed acylimidazole acylating reagents robustly and reproducibly modify RNA both inside and outside the cell. In contrast to existing reports, we provide direct evidence that 1M7 produces little or no detectable signal of RNA modification inside living cells. These surprising results are likely to have a far-reaching impact on how SHAPE experiments are conducted by the many laboratories that perform them.

## RESULTS AND DISCUSSION

We recently reported that acylimidazole SHAPE reagents FAI and NAI are able to modify RNA inside living cells, while an *N*-methylisatoic anhydride SHAPE reagent NMIA was not (Spitale et al. 2012). However, recent reports have suggested that 1M7, an isatoic anhydride reagent related to NMIA, was able to robustly modify both ribosomal RNA as well as mRNAs inside both mammalian and bacterial cells (McGinnis et al. 2015; Smola et al. 2015; Takahashi et al. 2016; Watters et al. 2016). These apparently contradictory results stimulated us to further investigate the differences between the two types of SHAPE reagents.

**FIGURE 2.**
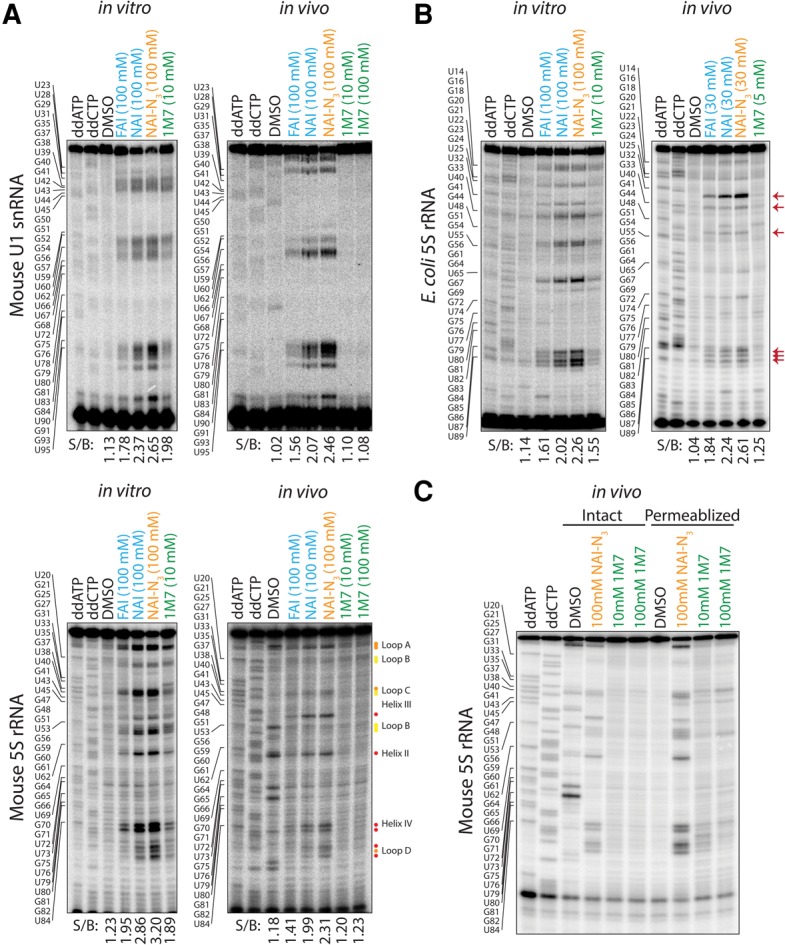
Analysis of cDNA truncations generated by various SHAPE electrophiles. (*A*) ^32^P radioblot comparing in vitro and in vivo modification levels of FAI, NAI, NAI-N_3_, and 1M7 on mouse U1 snRNA and 5S rRNA as measured by cDNA synthesis truncation and denaturing PAGE. Annotations of regions and flexibility as in Spitale et al. (2012). (*B*) Comparison of in vitro and in vivo modification levels of FAI, NAI, NAI-N_3_, and 1M7 on *Escherichia coli* 5S rRNA as measured by cDNA synthesis truncation and denaturing PAGE. Red arrows denote highly conserved accessible nucleotides between mouse and *E. coli* in vivo SHAPE experiments (Spitale et al. 2012). (*C*) Comparison of in vivo modification levels of NAI-N_3_ and 1M7 on intact and permeabilized mouse cells as measured by cDNA synthesis truncation of the 5S rRNA and denaturing PAGE.

Since the steps following SHAPE modification differ between methods using 1M7 or NAI-N_3_ (Fig. 1B), we reasoned that their ability to produce traditional cDNA truncations by primer extension would provide a basis for direct and unbiased comparison. We therefore used the traditional SHAPE method to compare the cDNA truncations generated by four SHAPE electrophiles: FAI, NAI, NAI-N_3_, and 1M7. RNA was either “in vivo” or “in vitro” modified; defined by adding the SHAPE electrophile directly to live cells or extracting RNA from cells first and then adding the electrophile to the RNA in a test tube, respectively. We first measured the structure of two abundant mouse RNAs, the U1 small nuclear RNA (snRNA) and the 5S ribosomal RNA (rRNA). All four electrophiles generated robust cDNA truncations for these RNAs in vitro (Fig. 2A). FAI was previously shown to be less reactive than NAI or NAI-N_3_ (Spitale et al. 2012), which we consistently observed in these experiments. Using synthesis and reaction conditions as published, 1M7 produces structure stops comparable to those produced by FAI; however, the precise stops are well correlated with the other reagents suggesting all four chemicals accurately read out RNA structure in vitro. A measure of signal-to-background (S/B) was obtained for each lane in the blot by comparison of the dynamic range of stops over the RNAs (Materials and Methods). Quantitative results were consistent with our observations that FAI and 1M7 produced comparable signal-to-background ratios, but revealed that 1M7 was slightly better than FAI in vitro. NAI and NAI-N_3_ samples produced the highest ratios of the four reagents (Fig. 2A). These results directly and clearly confirm that all tested SHAPE reagents read RNA secondary structure in vitro.

When assaying in vivo modified RNA, all but the 1M7 modified samples generated robust, high signal-to-noise cDNA truncation stops (Fig. 2A). The published acylation reagent concentration for “in vivo” 1M7 modification is 10 mM; however, to test whether adding excess reagent could overcome the lack of signal at the prescribed concentration, we also modified mouse ES cells with 100 mM 1M7. With a 10-fold increase in available 1M7, we observed similarly little modification as in the 10 mM condition (Fig. 2A). Quantitative measurements of the in vivo modified condition also showed that NAI and NAI-N_3_ had the greatest dynamic range. However, in this case, 1M7 signal-to-background ratios were not only lower than that of FAI, but also close to the S/B ratio of the unmodified DMSO sample, indicative of no signal over background (Fig. 2A). These results were again consistent across the assayed RNAs. Notably, these RNAs represent cytoplasmic (5S rRNA) and nuclear (U1 snRNA) localizations, demonstrating robust labeling with FAI and NAI derivatives across two cell membranes. These results overall demonstrate the acyl imidazole SHAPE reagents are robust RNA modifiers in living cells, whereas 1M7 is not. NMR analysis of the 1M7 and stock reagent before and after the experiments showed that the chemical was intact (not shown). Furthermore, experiments with in vitro modification were performed both during and after experiments with in vivo modification with successful in vitro RNA modification, so this cannot be attributed to 1M7 degradation.

Published work has reported in vivo modification of RNA in *E. coli* cells; however, no cDNA truncation blots were shown (McGinnis et al. 2015; Smola et al. 2015; Takahashi et al. 2016). To address the possibility that our lack of in vivo signal using 1M7 is due to the type of cells used, we repeated the SHAPE experiment in HST08 *E. coli* cells following precisely the published protocols for cell culture, RNA modification, and RNA extraction (McGinnis et al. 2015). As with the mouse samples, all four acylation reagents were successful at measuring RNA structure stops from in vitro modified RNA; however, only 1M7 was not able to generate robust data from live cell modification (Fig. 2B). Signal-to-background measurements in *E. coli* (Fig. 2B) were generally consistent with the results from the mouse cells, though 1M7 signal-to-background was slightly higher than that in the DMSO-treated control (but still less than one-fifth of the S/B over background of NAI). Together, these data suggest that 1M7 is considerably less effective at modifying RNA inside live cells when compared to FAI, NAI, and NAI-N_3_; approaching a level where there is little observable structure data as assayed by primer extension.

The above results demonstrate that 1M7 is unable to modify RNA to a level that is detectable by classic reverse transcription protocols. Although it is unlikely that there is robust modification that our experiments are not detecting, it is possible that very low levels of modification can occur that require deep sequencing to detect. Importantly, our in vitro experiments validate the proper and accurate chemical synthesis of 1M7 as an accurate SHAPE probe. While 1M7 has high temporal specificity for RNA dynamics in vitro, these results suggest that caution should be used when utilizing 1M7 for in vivo SHAPE RNA structure probing.

We considered possible origins of the poor cellular activity of 1M7. One is poor aqueous solubility due to low polarity, which could cause 1M7 to reach only low solution free concentrations and possibly become trapped in nonpolar membranes. Calculated logS values show ∼10-fold lower solubility for 1M7 (−2.4) as compared with NAI (−1.3) and NAI-N_3_ (−1.4). A major difference between the in vivo and in vitro conditions for SHAPE mapping are the cell walls and membranes encasing the transcriptomes of these organisms. We therefore hypothesized that lack of 1M7 signal from in vivo experiments comes from the impermeability of 1M7 to cell barriers, and therefore, permeabilization of these barriers would increase 1M7's acylation signal. To this end, we compared the SHAPE signal of mouse ES cells modified with NAI-N_3_ and 1M7 in “intact” and “permeabilized” conditions, effectively live and dead cells, respectively. Briefly, to permeabilize cells we incubated them in a dilute, nonionic detergent (0.05% NP-40) for 5 min at 25°C. While NAI-N_3_ samples produced comparable SHAPE signals between the intact and permeabilized conditions, 1M7 modified samples had increased signal after cell permeabilization (Fig. 2C). 1M7 modification on intact cells did not generate visible SHAPE stops, while modification on the permeabilized cells showed faint RT stop signals (Fig. 2C). These results are consistent with our hypothesis that 1M7 will tend to be limited by living cell barriers.

By implementing a traditional approach to interpreting SHAPE modification, we were able to directly compare the RNA acylation capacity of four SHAPE electrophiles on in vivo and in vitro modified RNA. FAI and NAI derivatives were specifically designed and synthesized to have properties amenable for labeling of RNAs inside living cells, such as longer half-lives and higher solubilities (Spitale et al. 2012). Comparison of these reagents to 1M7, which has a short half-life and relatively low aqueous solubility, revealed substantial deficits of 1M7's ability to modify RNA in vivo. These results were reproducible across multiple RNAs and multiple target cell types. Importantly, recent work from numerous laboratories has used various SHAPE reagents in vitro, with results validated by a number of orthogonal techniques (Somarowthu et al. 2015; Guo and Bartel 2016; Hawkes et al. 2016; Pirakitikulr et al. 2016). These published examples and our head-to-head comparison of SHAPE electrophiles provide clear and direct evidence that in vitro the reagents are largely equivalent. Electrophile choice for in vitro experiments can therefore be made based on desired reaction kinetics and ease of reagent accessibility.

Our strategy leverages radioisotopic labeling of RT primers and PAGE analysis. Such strategy is similar to published reports using reverse transcription and fluorescent reporters in capillary electrophoresis (Das et al. 2005; Wilkinson et al. 2008; Weeks 2010). More recent reports of using 1M7 for RNA structure analysis inside cells have relied on sequencing based approaches (Siegfried et al. 2014; McGinnis et al. 2015), in which library amplification would render the data perhaps more sensitive to the identification of SHAPE cDNA stops. In spite of this sensitivity difference, the reduced 1M7 signal in vivo raises two concerns, both in the raw data and the sequencing result. With respect to the raw data, the inability of 1M7 to modify a significant amount of RNA inside the cell membrane is worrisome because this suggests that the majority of structure data in 1M7-modified in vivo samples may come from cells with compromised cell membranes (i.e., dead cells), which are always present at low frequencies even in healthy cultures. This presents a problem because most applications of in vivo SHAPE require measurement of RNA structure in live, healthy cells. Several recent reports have demonstrated that after cell lysis, RNA molecules can reassociate with *trans*-acting RNAs and proteins (Mili and Steitz 2004; Riley et al. 2012; Riley and Steitz 2013), which would likely alter the RNA structures of such RNAs. Such reasons underscore the concern that 1M7 may be preferentially modifying RNAs in nonnative contexts.

Regarding sequencing, these results inform the relative efficiency of using 1M7 in sequencing-based methods for in vivo structure data. Even assuming all structure data is accurately measured, low modification efficiency will produce a low fraction of modified RNAs, which will in turn dramatically increase the background noise and the depth of sequencing required to obtain usable structure data. This greatly increases both time and cost of generating SHAPE results. These problems may be compounded by orders of magnitude for methods that rely on RT mutation, due to low mutation frequency, generation of indel mutations that cannot be uniquely mapped, and sequence contexts that could influence mutation rates (Siegfried et al. 2014). These effects can be seen in the overall coverage of the transcriptome and the number of PCR cycles required to generate a sufficient amount of sequencing library. There are ways to increase signal-to-noise, at the level of accurate structure stops. Background noise in SHAPE experiments comes from inadvertent RT stops or mutations, depending on the readout method. Selective purification of SHAPE-modified product is one strategy that reduces noise (Spitale et al. 2015); specifically this approach depleted bands that corresponded to background bands also present in the DMSO sample. Finally, reducing PCR amplification cycles will lower the chance of mutation introduction or “jackpotting” that make background noise appear like a signal.

As SHAPE and other molecular tools are further advanced and applied to different systems, it will become ever more important to have a precise understanding of the characteristics and specific limitations of each chemical tool. Our work here provides an initial but crucial characterization of two classes of frequently used SHAPE reagents. Novel chemical reagents and experimental conditions should be similarly compared to accurately determine which tool is best suited for the biological question at hand.

## MATERIALS AND METHODS

### SHAPE reagents

SHAPE reagents FAI and NAI were prepared as reported previously from 2-methyl-3-furoic acid and 2-methylnicotinic acid, respectively (Spitale et al. 2012). NAI-N_3_ was synthesized in four steps from ethyl 2-methylnicotinate analogously to the procedure described in Spitale et al. (2015). Reagent 1M7 was synthesized according to the published procedure from 4-nitroisatoic anhydride (Mortimer and Weeks 2007).

### Cell culture

V6.5 mouse embryonic stem cells were grown at 37°C with 5% CO_2_ in six-well plates coated with 0.2% gelatin. mESC growth medium was Knockout DMEM (Gibco) supplemented with 15% FBS, 1% PenStrep, 1% MEM NEAA, 1% GlutaMax, 0.2% β-mercaptoethanol, and 0.01% LIF. HST08 *E. coli* cells were grown in LB medium with ampicillin at 37°C.

### In vitro SHAPE modification

Mouse ES cell RNA was collected from cells by TRIzol and chloroform preparation, followed by RNeasy Mini column (Qiagen). *E. coli* RNA was extracted by bacterial lysis as stated in McGinnis et al. (2015), which consisted of an incubation in bacteria lysis buffer for 15 min on ice, followed by two freeze–thaws of the bacteria. Bacterial RNA was then purified from cells by TRIzol and chloroform preparation, followed by RNeasy Mini column (Qiagen). Bacteria lysis buffer was 20 mM HEPES-KOH (pH 7.8), 0.5 mM MgCl_2_, 100 mM NH_4_Cl, 4 mM β-mercaptoethanol, and 16% (wt/vol) sucrose supplemented with 15 µL of 50 mg/mL lysozyme.

Purified RNA from both mouse and bacteria cells was folded by a 95°C denaturation, a quick cool to 4°C, followed by 5 min incubation at 37°C in 100 mM HEPES pH 7.5, 6 mM MgCl_2_, and 100 mM NaCl. Samples were then modified for 5 min at 37°C with either the DMSO control or the SHAPE reagents FAI, NAI, NAI-N_3_ at 100 mM final or 1M7 at 10 mM final. Modified RNA was then cleaned up and eluted in pure, RNase-free water using RNeasy Mini columns (Qiagen) before primer extension.

### In vivo SHAPE modification

RNA was modified and collected from mESCs using the same conditions as previously published for mammalian cell modification (Smola et al. 2015). Old media was removed and cells were washed once with PBS. Of note, 900 µL of fresh medium was replaced on the cells. One hundred microliters of 10× SHAPE Chemical in DMSO, or pure DMSO, was then added to each 900 µL sample at the following final concentrations: 100 mM FAI, 100 mM NAI, 100 mM NAI-N_3_, 10 mM 1M7, and 100 mM 1M7. Cells were modified for 5 min at 37°C. Media were removed, and RNA was purified by TRIzol and chloroform preparation followed by RNeasy Mini Column (Qiagen).

*E. coli* cells were modified with SHAPE reagent using the conditions as written in McGinnis et al. (2015). Cells in LB medium were modified for 5 min at 37°C with SHAPE reagent dissolved in anhydrous DMSO. Final concentrations of SHAPE reagents were as follows: FAI, NAI, NAI-N_3_ at 30 mM and 1M7 at 5 mM, all with a final DMSO concentration of 3% (v/v) in LB. Modified RNA was extracted from bacteria by lysis as described earlier, followed by TRIzol chloroform preparation and RNeasy Mini column (Qiagen).

### RT primers

Mouse U1 snRNA: 5′-CCCACTACCACAAATTATGCAG-3′
Mouse 5S rRNA: 5′-AAAGCCTACAGCACCCGGTAT-3′
*E. coli* 5S rRNA: 5′-TGCCTGGCAGTTCCCTACTC-3′

### Primer extension and PAGE

RT primers were synthesized by IDT, with basic desalting purification, and then radiolabeled with ^32^P using OptiKinase and [γ-^32^P]–ATP. RNA was reverse transcribed into cDNA with SuperScript III at 55°C for 25 min. After cDNA synthesis, the remaining template RNA was degraded by adding NaOH and heating for 5 min at 95°C. The samples were then run across a 35-cm-long 8% Urea PAGE gel. The gel was dried for 2 h on a vacuum gel drier and then exposed for 24 h.

### Signal-to-background ratio calculation

The signal-to-background (S/B) ratio reported for each lane is the arithmetic mean of the ratios (Signal/Background) of three bands. Signal is defined as mean pixel intensity of a band as measured by ImageJ (Analyze > Measure). Background is defined as mean pixel intensity of a low intensity region next to the signal band. The bands were in the same position for all lanes within a blot and were chosen based on presence in the greatest number of SHAPE modified condition lanes (i.e., FAI, NAI, NAI-N3, 1M7-modified), but not in the unmodified (i.e., DMSO) condition.
